# Retropharyngeal Lipomatous Hamartoma: Case Report and Review of Literature

**DOI:** 10.22038/IJORL.2022.64205.3197

**Published:** 2023-01

**Authors:** Sunil Sam Varghese, Ashish Varghese, Preethi-Anni-Mercy Paul

**Affiliations:** 1 *Department of ENT, Christian Medical College, Ludhiana, Punjab, India.*; 2 *Department of Pathology, Christian Medical College, Ludhiana, Punjab, India.*

**Keywords:** Hamartoma, Retropharynx, Lipoma, Stridor, Trans-oral excision

## Abstract

**Introduction:**

Lipomas of the of the head and neck region are rare, more so in the retropharyngeal space. Lipomas in this region can produce symptoms that demand surgical excision. This paper describes a case of lipomatous hamartoma of the retropharynx, which to the best of our knowledge has not yet been reported in English literature.

**Case Report:**

A 53-year-old gentleman presented to the ENT department with snoring, voice change and stridor. Examination revealed a smooth bulge in the posterior wall of the oropharynx causing near complete obstruction of the airway. A contrast enhanced computed tomogram revealed a non-enhancing hypodense lesion in the retropharyngeal space extending from C1-C4 level, which was suggestive of a lipoma. The tumour was surgically excised trans-orally. A limited review of literature is also presented.

**Conclusion:**

Trans-oral approach is preferred to external approach for surgical removal of benign retropharyngeal tumours that cause obstructive symptoms, as our case. This approach is safe, effective, and associated with lesser post-operative morbidity.

## Introduction

Lipomas are benign encapsulated tumours consisting of lobulated, slow growing mature adipocytes with minimal connective tissue stroma (1). Hamartomas are benign masses comprised of an admixture of cells and tissues indigenous to that site. Though initially thought to be developmental, these are now considered clonal due to the identification of chromosomal aberrations (2).

The retropharyngeal space is bounded on either side by the major neck vessels and lower cranial nerves making it a difficult region to gain access through the external approach. The trans-oral approach possesses the inherent advantage of avoiding the important neurovascular structures and lower post-operative morbidity (3). However, this vantage point is negated by the limited exposure and the compact narrow operating space available for manipulation with conventional instruments. Here we describe the clinical presentation of a 53-year-old gentleman with a retropharyngeal lipomatous hamartoma and its surgical management, which to the best of our knowledge has not yet been reported in the English literature. We had reviewed the PubMed database using the terms retropharynx and lipoma from the year 2005 till 2022. Only those case reports which provided information on the size, extent and surgical management were included in the review (Table.1).

**Table 1:** Review of the English language reported cases of retropharyngeal lipomas from the year 2005 to 2022

**Serial No.**	**Authors**	**Size of lipoma(cm)**	**Superior extent**	**Inferior extent**	**Parapharyngeal** **extension**	**Surgical approach**
1	Ghammam M et al. (1)	7.3X2.6X11.1	C2	C7	yes	External
2	Aydin U et al. (3)	12X7	C2	C6	no	Trans-oral
3	Namyslowski G et al. (4)	11.7X4.2X7.5	oropharynx	superior mediastinum	yes	External
4	Radhakrishna Pillai OS et al. (5)	8X5X11	C1	C7	no	Trans-oral
5	Chua DY et al. (6)	9.4X6.7	C1	C6	yes	Trans-oral
6	Piccin O et al. (7)	5X2X2.5	skull base	C3	no	Trans-oral
7	Heaton CM et al. (8)	4.4X2.3X1.3	C1	C3	no	TORS^#^
8	Lee HK et al. (9)	10X5X11	soft palate	thoracic inlet	yes	External
9	Jin SM et al. (10)	3.3X4	C1	C3	no	Trans-oral
10	Chrysovitsiotis G et al. (11)	4.5X3X1.5	Soft palate	Valleculae	no	Trans-oral
11	Luczak K et al. (12)	8.5X5.8X7.2	Skull base	Hyoid bone	yes	External

Legends to Table1, C- Cervical vertebrae. # - TORS: Trans oral robotic surgery

## Case Report

**Fig 1:**
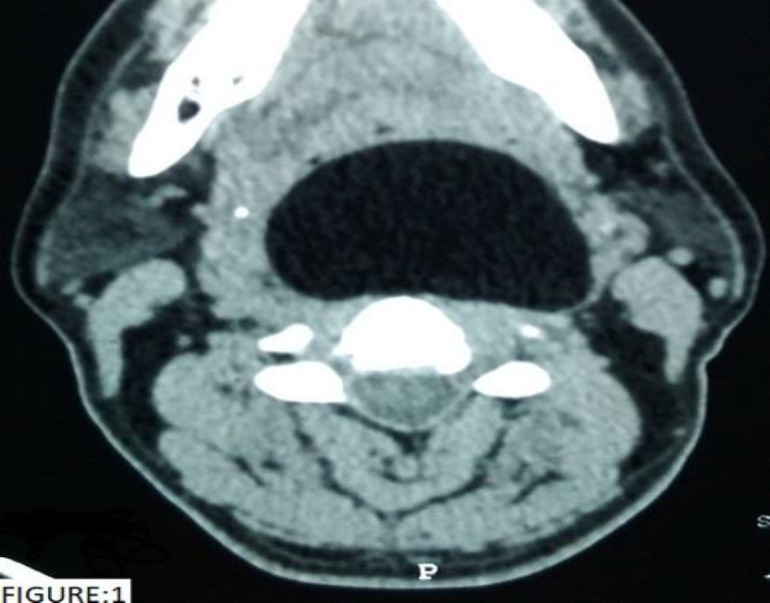
Axial section of the CECT neck showing a well-defined non enhancing fat density lesion severely compromising the oropharyngeal airway and displacing the carotid vessels laterally

**Fig 2:**
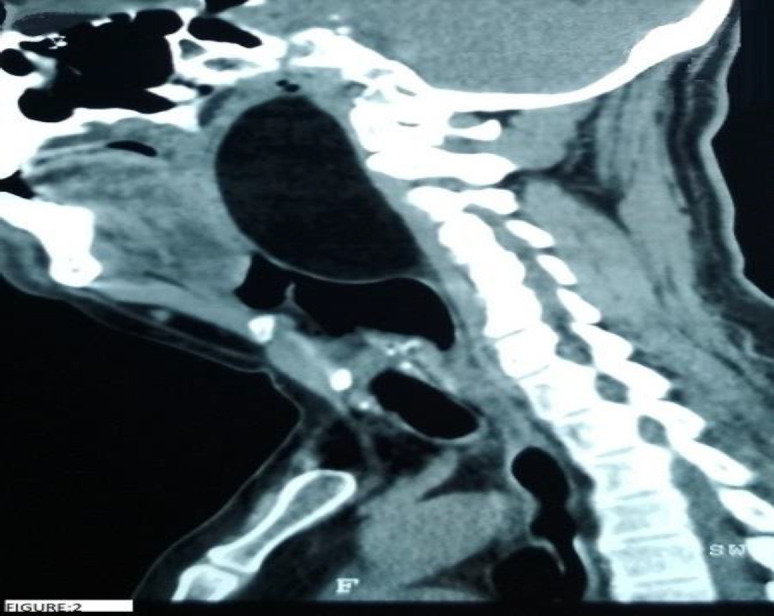
Cect Neck Sagittal Section- Lipoma Is Seen Extending From The Skull Base To Level Of C5

A 53-year-old gentleman with no co-morbidities presented to the outpatient department with complaints of noisy breathing for 20 days. He also gave history of progressive snoring and change in voice from 6 months. Examination revealed a smooth mass in the oropharynx extending inferiorly from the level of the base of tongue and superiorly beyond the level of the soft palate causing near complete obstruction of the oropharyngeal airway. A contrast enhanced computed tomogram (CECT) of the neck was done which was suggestive of a well-defined, non-enhancing fat density lesion in the retropharyngeal space measuring 8.2 cm craniocaudally, 4 cm antero-posteriorly and 6.2 cm transversely causing marked narrowing of the airway. The lesion is seen to compress the left carotid artery and displacing it laterally (Fig1). It was seen extending from the base of skull to the level of C5 vertebrae (Fig2).

As the patient was in stridor he was immediately shifted to the operation theatre where he underwent a tracheostomy under local anaesthesia. Tracheostomy secured the airway and relieved the respiratory distress. A trans-oral core biopsy of the lesion was done in the same sitting which was reported as a lipoma and the patient was planned for trans oral excision under general anaesthesia. 

**Fig 3:**
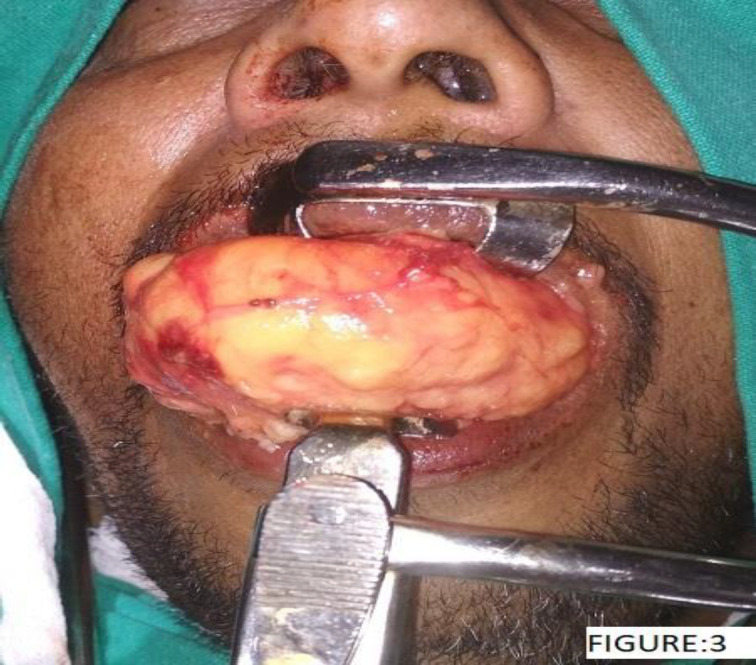
Delivering the Retropharyngeal Lipoma in Toto through the Oral Cavity

Patient was placed in the Rose position. Exposure of the lipoma was achieved using a Boyle Davis mouth gag. The soft palate was retracted to expose the superior limit of the tumour. A vertical incision was made over the posterior pharyngeal wall and the constrictor muscles exposing the lipoma. Dissection was carried out from above downwards with traction over the tumour, releasing its attachments by blunt dissection. The tumour was found anterior to the prevertebral muscles within the confines of the retropharyngeal space. The tumour was delivered per-orally as a whole (Fig 3). 

**Fig 4:**
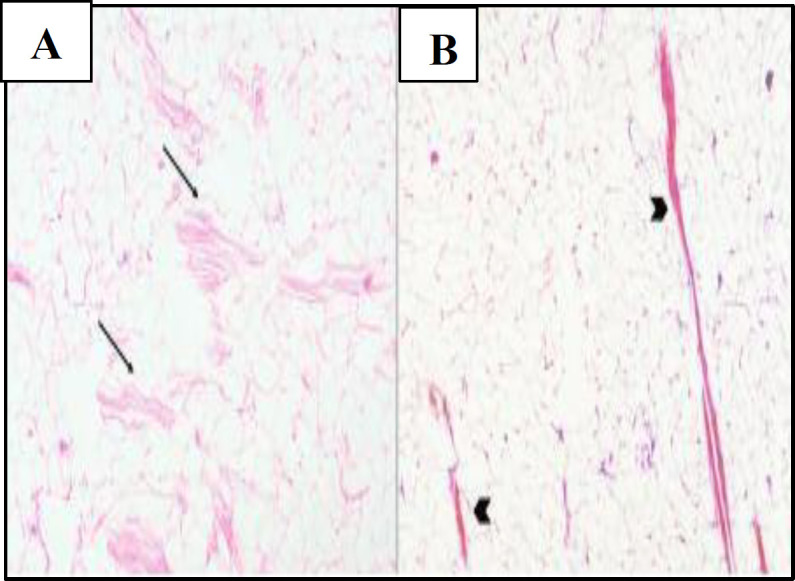
Lipomatous Hamartoma of the Retropharynx Showing **A**) Lobules of Mature Fibroadipose Tissue Separated By Skeletal Muscle Fibers (Black Arrows), Hematoxylin and Eosin, 100x; **B**) Skeletal Muscle Fibers Highlighted Red By Masson’s Trichrome Stain (Arrowheads), 100x

Gross examination revealed a partly encapsulated tumour measuring 8x6x3.5cm with yellow fatty areas on cut section. Histopathological examination revealed a lipomatous hamartoma, which showed lobules of mature adipose tissue with skeletal muscle bundles, few nerves and fibrocollagenous tissue (Fig.4 A-B). Post-operative period was uneventful, the tracheostomy tube was decannulated on post operative day three. Patient was kept on Ryles tube feeds for a week. 

## Discussion

Clinical presentation of benign retropharyngeal tumours depends on its location and size, it varies from a vague globus sensation, dysphagia, snoring with or without obstructive sleep apnoea syndrome or stridor with respiratory distress (4) or can be asymptomatic (13). Thirteen percent of lipomas occur in the head and neck region, and pharyngeal involvement is rare (5). Owing to the slow rate of growth, they are usually detected when the lipoma has reached a considerably large size (4) as may be the case here when snoring would have manifested years after the lipoma had appeared. On computed tomograms lipomas appear as homogenous hypodense lesions with attenuation varying from -50 to -150 Hounsfield units and does not enhance on contrast (1).

The pre-operative histological diagnosis was lipoma and the definitive treatment for a retropharyngeal lipoma is surgical excision (3,4). In this case, the lesion being well encapsulated was easily dissected out but its anatomical location posed a challenge with respect to access and exposure. 

The retropharyngeal space is in close proximity to major neurovascular structures on either side making the external approach to this space fraught with potential serious complications (1,6). The trans - oral route adopted in this case may seem straight forward and simple. However, due to limited exposure and small compact working space make surgical manipulations very cumbersome and difficult. The exact location of the lipoma determines the surgical approach. 

A significant lateral extension would warrant an external transcervical approach. Transoral excision of retropharyngeal lesions with parapharyngeal extension can be attempted, but visualisation of the lateral component of the tumour is compromised and dissection is done blindly with the finger (6). 

In our review of literature, all the patients that underwent an external surgical approach had parapharyngeal extension and two cases with parapharyngeal extension were removed trans-orally. The largest dimension among the lipomas removed trans-orally and externally in our review were 12 cm and 11.7 cm respectively. The nasopharyngeal part of the lipoma in this case was visualised after retracting the soft palate anteriorly. The soft palate can also be split in midline to gain access to this part of the tumour (7). In our review the inferior most extent of the lipomas that were removed trans-orally and externally were C7 vertebrae level and superior mediastinum respectively. Our review showed that the superior most extent of the lipomas removed trans-orally and externally was the level of the skull base. The more recent trans oral robotic surgery technology have added better visualisation, magnification and dexterity within the oropharynx (8). Owing to the presence of skeletal muscle tissue and few nerve bundles on histopathology, the diagnosis of lipomatous hamartoma was suggested. Lipomatous hamartomas are rare, and have been reported at various sites like the retroperitoneum, subcutaneous sites, and the orbit (14). However, to the best of our knowledge, no similar case has been reported in the English literature.

## Conclusion

Lipoma in the retropharyngeal space is very rare and to the best of our knowledge, this is the first case reporting of lipomatous hamartoma of the retropharynx. Complete surgical excision is curative. This case report shows that the trans oral route for surgical excision of benign retropharyngeal tumours such as a lipomatous hamartoma is a safe, feasible, and an effective option. The external approach provides better access but is associated with higher -operative morbidity.
